# Germline FFPE inherited cancer panel testing in deceased family members: implications for clinical management of unaffected relatives

**DOI:** 10.1038/s41431-021-00817-w

**Published:** 2021-03-02

**Authors:** Sarah Bennett, Elizabeth Alexander, Harry Fraser, Naomi Bowers, Andrew Wallace, Emma R. Woodward, Fiona Lalloo, Anne Marie Quinn, Shuwen Huang, Helene Schlecht, D. Gareth Evans

**Affiliations:** 1 Clinical Genetics Service, Manchester Centre for Genomic Medicine, St Mary’s Hospital, Manchester University NHS Foundation Trust, Manchester, UK; 2 Northern Regional Genetic Service, Genetics Health Service New Zealand, Auckland City Hospital, Auckland, New Zealand; 3 North West Genomic Laboratory Hub, Manchester Centre for Genomic Medicine, St Mary’s Hospital, Manchester University NHS Foundation Trust, Health Innovation Manchester, Manchester, UK; 4 Division of Evolution and Genomic Sciences, School of Biological Sciences, Faculty of Biology, Medicine and Health, University of Manchester, Health innovation Manchester, Manchester, UK; 5 Department of Anatomic Pathology, University Hospital Galway, Galway, Ireland

**Keywords:** Cancer genetics, Cancer genetics, Genetic counselling, Cancer genetics, Genetic testing

## Abstract

Where previously, germline genetic testing in deceased affected relatives was not possible due to the absence of lymphocytic DNA, the North-West-Genomic-Laboratory Hub (NWGLH) has developed and validated next-generation sequencing based gene panels utilising formalin-fixed-paraffin-embedded (FFPE) tissue DNA from deceased individuals. This technology has been utilised in the clinical setting for the management of unaffected relatives seen in the Clinical Genetics Service (CGS). Here we assess the clinical impact. At the time of data collection, the NWGLH had analysed 180 FFPE tissue samples from deceased affected individuals: 134 from breast and/or ovarian cancer cases for germline variants in the *BRCA1/BRCA2* genes and 46 from colorectal, gastric, ovarian and endometrial cancer cases for germline variants in a panel of 13 genes implicated in inherited colorectal cancer and gastric cancer conditions. Successful analysis was achieved in 140/180 cases (78%). In total, 29 germline pathogenic/likely pathogenic variants were identified in autosomal dominant cancer predisposition genes where the gene was pertinent to the cancer family history (including *BRCA1/BRCA2*, the mismatch-repair genes and *APC*). Of the 180 cases, the impact of the result on clinical management of unaffected relatives was known in 143 cases. Of these, the results in 54 cases (38%) directly impacted the clinical management of relatives seen by the CGS. This included changes to risk assessments, screening recommendations and the availability of predictive genetic testing to unaffected relatives. Our data demonstrate how FFPE testing in deceased relatives is an accurate and informative tool in the clinical management of patients referred to the CGS.

## Introduction

In the United Kingdom (UK), recommendations for the care of individuals with a family history of breast cancer are detailed in the National Institute for Health and Care Excellence (NICE) guidance [1]. Guidelines for the management of individuals with a family history of colorectal cancer have been produced by the British Society of Gastroenterology/Association of Coloproctology of Great Britain and Ireland/United Kingdom Cancer Genetics Group [2]. For individuals meeting local referral criteria, referrals to Clinical Genetics Services are made either by general practitioners, family history clinics, surgical teams or oncology teams. Regional Genetics Services provide genetic counselling, risk assessments, screening recommendations and in some cases also arrange for genetic testing in families where an inherited cause of cancer is suspected.

In the UK, diagnostic germline genetic testing of the *BRCA1/BRCA2* (hereafter *BRCA1/2*) genes is routinely offered through the National Health Service (NHS) to patients with a personal history (and in some cases a wider family history) of breast and/or epithelial ovarian cancer. At the point data for this article was collated, genetic testing was recommended when a family history or personal tumour pathology suggested there was at least a 10% chance of a pathogenic/likely pathogenic variant in one of these genes being identified [1]. Throughout the Genetics Services in the UK, this probability is generally calculated using the pathology-adjusted Manchester Scoring system [3] and/or the BOADICEA risk model [4]. Genomic testing in England is currently undergoing a restructure of services and it is likely the threshold for testing will be lowered to at least a 5% detection rate in the near future.

For genes associated with inherited colorectal and gastric cancer conditions (including Lynch syndrome, Familial Adenomatous Polyposis, MUTYH-Associated Polyposis and Hereditary Diffuse Gastric Cancer), diagnostic testing is available through UK Regional Genetics Laboratories to affected individuals where an inherited cause is suspected [2, 5]. Guidelines for testing vary depending on the condition suspected. In the case of Lynch syndrome for example, genetic testing is usually offered to affected individuals whose personal and family history meet the modified Amsterdam criteria or whose tumour demonstrates abnormal immunohistochemistry without evidence of MLH1 promoter hypermethylation and/or BRAF p.V600E pathogenic variant [2].

When considering genetic testing in families where an inherited cause of cancer is suspected, it is most informative to offer germline genetic testing to affected individuals. This is because if a pathogenic variant is present in the family, it is these individuals in whom it is most likely to be detected. In some families, where all affected relatives are deceased, genetic testing can be offered to unaffected individuals, often referred to as ‘indirect’ or ‘unaffected’ testing [1]. However, negative test results in this context are often uninformative, as in most cases, a negative result cannot exclude an increased risk of a site-specific cancer in the individual tested or their relatives. There are several reasons for this: it may be that an inherited cause of cancer is present in the family but the ‘unaffected’ individual tested has not inherited the familial gene variant; it may be that an inherited cause of cancer is present in the family but is due to a variant in a different gene or combination of genes not tested for; or it may be that the cancers in the family are not due to an underlying inherited cause.

Another type of testing has been developed over the last decade, whereby DNA extracted from stored tissue samples from a deceased individual can be tested for variants in specific genes [6–11]. It is common practice for malignant and healthy tissue samples taken at biopsy or surgery to be immersed in formalin to preserve the specimen for pathological examination [12]. These samples are typically stored in paraffin blocks and retained for a period of 30 years. Historically, genetic testing using DNA extracted from formalin-fixed-paraffin-embedded (FFPE) tissue has been challenging due to the quality of DNA preserved [6]. Over the last decade, technical advances in the use of FFPE samples as a source of germline DNA have progressed from testing for specific founder pathogenic variants to conducting next-generation sequencing (NGS) panels [7–11].

The North-West Genomic-Laboratory Hub (NWGLH) has developed a method for performing germline *BRCA1/2*, mismatch repair (MMR), polyposis and inherited diffuse gastric cancer related genetic testing in archived pathology tissue samples from deceased index cases. This is performed using DNA extracted from FFPE tissue. Two NGS panels have been optimised for the detection of point mutations and small-scale genetic/genomic insertion/deletions [7] and in early 2016, this technology was translated into the clinical setting. Using this technology has allowed informative genetic testing in families where this had not previously been possible in the absence of living affected relatives. The NWGLH is a UK accredited laboratory [13] and participates in the UK National External Quality Assessment (NEQAS) scheme [14]. Here, we present data from the initial 180 FFPE germline NGS panel tests performed by the NWGLH as part of the NHS clinical service. We include the methodology of the laboratory techniques and the clinical impact of these results on the management of unaffected relatives.

## Methods

**Fig. 1:**
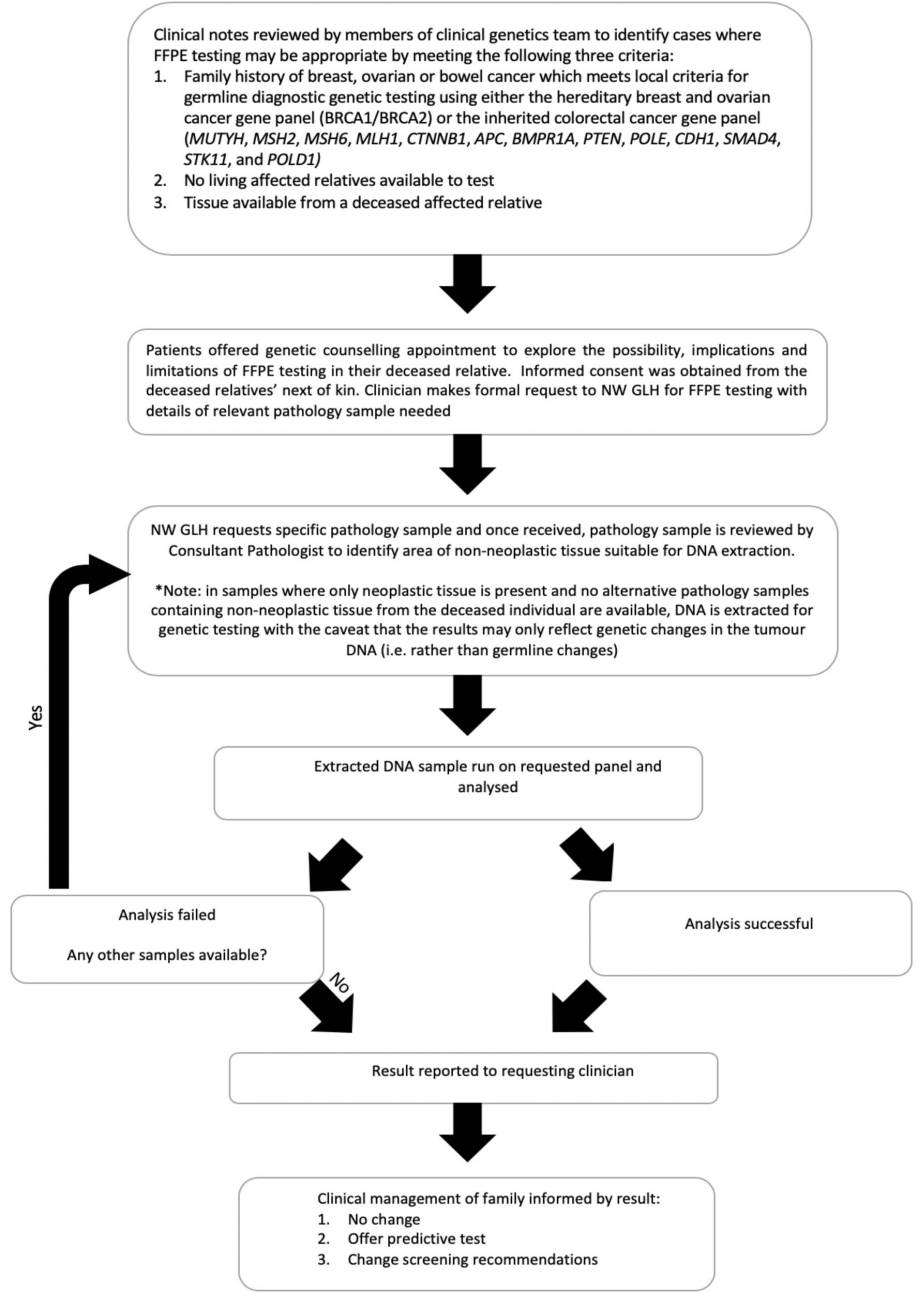
Flow chart of process followed for identifying and managing families where FFPE testing in deceased relatives could be offered.

Cases in which FFPE testing could be utilised were initially identified by Clinical Geneticists and Genetic Counsellors during the routine review of cases by the Manchester Clinical Genetics Service. This process is set out in Fig. 1. As part of the clinical assessment of a family history, it is routine practice to request histology information of certain diagnoses in the family. Cases meeting the established criteria were identified and patients previously seen by our service were offered a clinical genetics appointment to explore the possibility, implications and limitations of FFPE testing in their deceased relative. Informed consent was obtained from the deceased relatives’ next of kin, as per the Human Tissue Act [15], and requests for FFPE genetic testing were passed to the NWGLH using their specific request form.

Ideally, non-neoplastic tissue samples were requested but where this was not possible, samples containing neoplastic material were obtained. These samples then underwent pathology assessment by light microscopy of a haematoxylin and eosin (H&E) stained slide to confirm they comprised of non-neoplastic tissue with no evidence of malignancy. Where a block of non-neoplastic tissue was not available, an area of tissue uninvolved by tumour was marked on a H&E guide slide by a Consultant Pathologist. These samples typically originated from resection or surgical excision specimens which facilitated the selection of a non-neoplastic area on a H&E-stained slide. This ensured that any variant identified was likely to represent a germline variant. If no useable tissue was identified, this was fed back to the clinical team and families informed that the test could not be carried out. Only cases where tissue for testing were received are included in this report. All sample handling and processing was conducted following standard operating procedures (SOPs) in a clinical laboratory accredited to ISO15189. As such, sample transfers in SOPs are subjected to a procedure specific risk assessment and control measures appropriate to the overall risk. Example control measures employed include witness checks, self checks and tube order checks.

In each case analysed, four x 5-µM-thick FFPE sections were processed to extract genomic DNA using the Roche cobas^®^ DNA Sample Preparation Kit. Double stranded DNA was quantitated using a Qubit fluorometer and samples normalised to 5 ng/µL concentration. Library enrichment used a short amplicon approach using the Qiagen GeneRead DNAseq *BRCA1/2* v2 kit and a 13 gene colorectal cancer custom design using a Qiagen GeneRead DNAseq Custom Panel v2. The colorectal cancer panel includes *MUTYH*, *MSH2*, *MSH6*, *MLH1*, *CTNNB1*, *APC*, *BMPR1A*, *PTEN*, *POLE*, *CDH1*, *SMAD4*, *STK11*, and *POLD1*. Analysis of PMS2 using this technology was not feasible due to the presence of multiple highly homologous pseudogenes. Library enrichment comprised 250 primer pairs split between 4 multiplex primer pools for *BRCA1/2* and 8 multiplex primer pools for colorectal cancer. Twenty nanograms of normalised FFPE-derived DNA was amplified in each multiplex primer pool. Hundred percent of the *BRCA1/2* coding regions and immediate intron exon boundaries are covered by the *BRCA1/2* v2 enrichment. For the colorectal cancer custom panel, the percentage of coding regions and immediate intron exon boundaries covered by the enrichment of key genes were as follows: *MLH1* 100%, *MSH2* 99.99%, *MSH6* 99.99%, *APC* 99.99%, *CDH1* 99.98%. 100× vertical coverage was regarded as the minimum depth for variant calling. In order to be classed as a successful analysis, a minimum 97% coverage at 100× depth over the region of interest was required. The mean vertical coverage of most samples successfully analysed was in excess of 1000×.

Following PCR-based target enrichment, library preparation and purification followed a custom protocol using AMPure XP beads from Agencourt for size selection and Illumina TruSeq PCR Free indexes and reagents for indexing. The DNA library was then paired end sequenced on an Illumina MiSeq with v2 chemistry (2 × 150 cycles). Bioinformatic analysis used an in-house developed bioinformatics analysis pipeline which was validated to detect low-level mosaic calls down to 4% allele fraction and used a software consensus between VarScan v2.3.6 and DREEP v0.7. Large indel events were assessed using Pindel (v0.2.4.t). In addition, an assessment was made of the frequency of each variant called within each batch of samples analysed. This aided in the interpretation of whether a variant was real or an artefact. Variants identified bioinformatically were assessed for clinical relevance following American College of Medical Genetics and Association for Clinical Genomic Science guidelines [16, 17] on variant interpretation. All pathogenic variants, likely pathogenic variants and variants of unknown significance (Classes 5, 4, and 3, respectively) were confirmed by Sanger sequence analysis (BigDye v3.1 chemistry). As per the routine practice, all cancer predisposition testing data were submitted to Public Health England who make the data freely available via the CanVarUK database (available at: www.canvaruk.org).

The results of FFPE genetic testing were reported to the requesting clinician who then communicated the results to patients. If genetic analysis was successful, assessments of the family history were reviewed and any changes to screening recommendations or management options were documented. Data regarding the clinical impact of the results of FFPE testing was collected by a team of three Genetic Counsellors. Reports were available for the 180 tests done between May 2016 and September 2018. Of these, 113 tests were requested by the clinical genetics team in Manchester. Using a systematic approach, the clinical files for each of these 113 cases were reviewed to determine the impact of the FFPE genetic testing result on the management of relatives seen by our clinical service. For the 67 tests requested by external Clinical Genetics Services, the impact of the result on clinical management of relatives could only be measured when the outcome was clear from the result i.e. a pathogenic/likely pathogenic variant was identified or in cases where analysis failed.

## Results

**Fig. 2:**
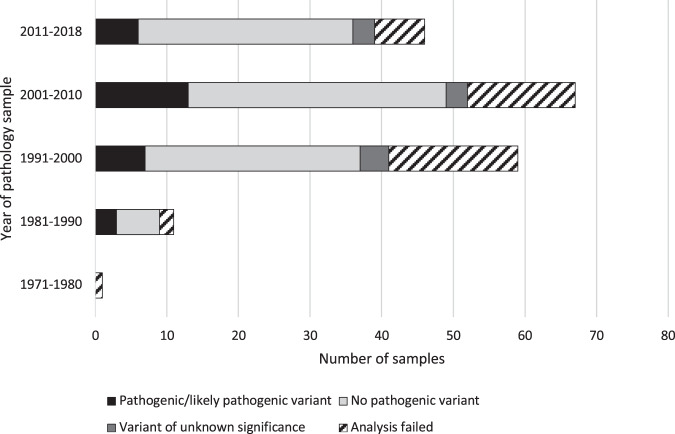
Results of FFPE analyses based on age of pathology samples—BRCA1/2 panel and inherited colorectal panel analyses combined.

Of the 180 FFPE samples tested, 134 were *BRCA1/2* panel analyses and 46 were inherited colorectal cancer panel analyses. For the purpose of this paper, the results are recorded as either: pathogenic/likely pathogenic variant identified (i.e., Class 5 and Class 4 variants), variant of unknown significance identified (i.e., Class 3), no variant reported (Class 1, Class 2 or no variant identified), or analysis failed. Table 1 details the results and the recorded diagnoses in each of the 180 samples tested along with the results and neoplastic content of the tissue samples used, for both the *BRCA1/2* analyses and the inherited colorectal panel analyses, respectively. The age of the pathology sample used was also recorded for all 180 samples and is detailed in Fig. 2 with the corresponding result. Successful analyses were achieved from samples dating back to 1986.

**Table 1:** Results of FFPE panel analyses looking at original diagnosis in index case and the type of tissues tested.

		Index patient diagnosis	Pathogenic/likely pathogenic variant	No variant reported	Variant of unknown significance	Analysis failed
FFPE panel used	BRCA1/2 panel	Breast cancer	4	23	2	12
		Bilateral breast cancer	1	11	2	1
		Ovarian/fallopian tube/peritoneal cancer	10	44	1	9
		Breast & ovarian cancer	4	5	1	2
		Male breast cancer	1	0	0	1
	Inherited colorectal panel	Bowel cancer	8	15	1	12
		Ovarian cancer	0	1	1	3
		Endometrial	0	1	0	0
		Gastric cancer	0	2	1	0
		Familial Adenomatous Polyposis	1	0	0	0
Tissue type used		Non-neoplastic (*n* = 105)	22	50	8	25
		Neoplastic content <20% (*n* = 7)	3	3	0	1
		Neoplastic content 20–50% (*n* = 9)	1	7	0	1
		Neoplastic content >50% (*n* = 21)	1	17	0	3
		Neoplastic content not specified (*n* = 15)	2	8	0	5
		Tissue type not specified on report, i.e., neoplastic content unknown (*n* = 23)	0	17	1	5

### Results and clinical impact of FFPE BRCA1/2 panel analyses

**Fig. 3:**
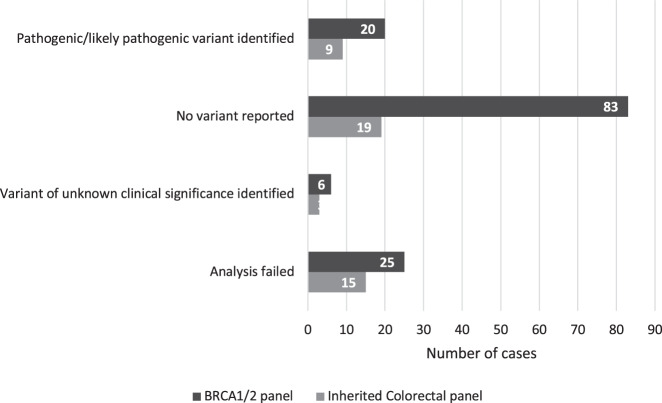
Overview of test results of BRCA1/2 and Inherited colorectal panel analyses.

**Fig. 4:**
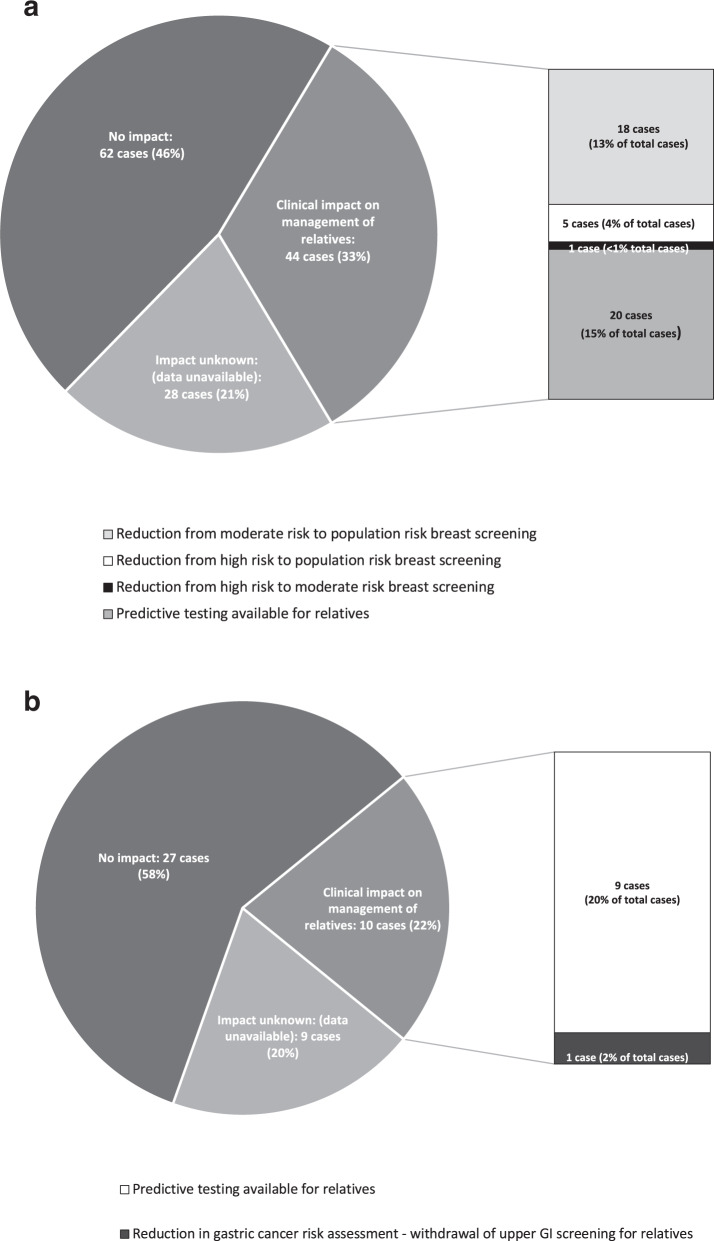
Impact of FFPE panel results on clinical management of family members. **a** Impact of all 134 FFPE BRCA1/2 panel results **b** Impact of all 46 FFPE inherited colorectal panel results.

As depicted in Fig. 3, of the 134 samples tested on the *BRCA1/2* panel, analysis was successful in 109 samples (81%). In 15% of total cases, a pathogenic or likely pathogenic variant was identified. No variant was reported in 62% of cases and a variant of unknown clinical significance was identified in 4% of cases. Analysis failed in 19% of cases. Fig. 4a details the impact on the clinical management of relatives in the 134 cases tested using the *BRCA1/2* panel. For the 25 cases in which the *BRCA1/2* analysis failed, the results will not have impacted management of relatives as advice will remain the same as prior to the test being offered.

Of the 109 successful *BRCA1/2* panel analyses, 71 were requested by the Manchester Clinical Genetics Service, and 38 were requested by external Clinical Genetics Services. For the Manchester cases, the impact of a successful analysis on the clinical management of relatives was assessed in all 71 cases. Of those requested by external centres, the impact on clinical management was only known (as this could be assumed) in the ten cases where a pathogenic/likely pathogenic variant was identified. Therefore, in total the impact of successful FFPE *BRCA1/2* panel analyses on the clinical management of relatives could be determined in 81 cases. Of this group, the results of testing changed the clinical management of relatives in 44 cases (54%). In the 20 cases where a pathogenic/likely pathogenic variant was identified, predictive genetic testing then became available to the deceased individual’s family members. See Supplementary Table 1 for details about these variants. In 24 cases, a negative result (i.e., no *BRCA1/2* variants reported) affected the breast cancer risk assessment and screening recommendations provided for family members. In some of these families, women previously assessed to be at a ‘high risk’ of breast cancer based on their family history information were then reassessed as either ‘moderate risk’ or similar to population risk. Taking all 134 cases and each possible result into account (ie. both successful and failed analyses), offering FFPE *BRCA1/2* panel testing affected clinical management of relatives in at least 33% of cases. We estimate that this figure is likely to be higher than this, as we are unable to assess the impact of these results on the clinical management of relatives for the majority of tests requested by external Clinical Genetics Services.

### Results and clinical impact of FFPE Inherited colorectal cancer panel analyses

As depicted in Fig. 3, of the 46 samples tested on the inherited colorectal cancer panel, analysis was successful in 31 samples (67%). In 20% of these cases, a pathogenic or likely pathogenic variant was identified in a pertinent cancer susceptibility gene. No variant was reported in 41% of cases and a variant of unknown clinical significant was detected in 6% of cases. Analysis failed in 33% of cases. Fig. 4b details the impact on the clinical management of relatives in the 46 cases tested using the inherited colorectal cancer panel. For the 15 cases in which the analysis failed, the results did not impact management of relatives as advice remained as it was prior to FFPE testing.

Of the 31 successful colorectal panel analyses, 20 cases were requested by the Manchester Clinical Genetics Service, and 11 by external Clinical Genetics Services. For the Manchester cases, the impact of a successful analysis on the clinical management of relatives was assessed in all 20 cases. Of those requested by external services, the impact on clinical management was only known (as this could be assumed) in the two cases where a pathogenic/likely pathogenic variant was identified. Therefore, in total the impact of successful FFPE inherited colorectal panel analyses on the clinical management of relatives could be determined in 22 cases. Of this group, the results of testing changed the clinical management of relatives in ten cases (45%). In the nine cases where a pathogenic/likely pathogenic variant was identified, predictive genetic testing then became available to the deceased individual’s family members. See Supplementary Table 1 for details about these variants. In one case, the absence of a pathogenic/likely pathogenic CDH1 variant impacted gastric cancer screening recommendations for relatives. Of note, in this case a VUS was found in another gene and so the file remains on review. Taking all 46 cases and each possible result into account (i.e., successful or failed analysis), offering FFPE inherited colorectal cancer panel testing affected clinical management of relatives in at least 22% of all cases.

## Discussion

In this cohort, where testing a deceased affected family member was previously not possible, successful analysis was achieved in 140 out of the total 180 cases (78%). In at least 54 of the total cases (30%), the results impacted the clinical management of living relatives.

Successful analysis was possible from samples dating as far back as 1986, so interestingly, the success of testing did not appear to be dependent on the age of the tumour tissue but was more likely related to the quality of fixation of the pathology sample. It is widely accepted that fixation conditions are a strong determinant of the quality of nucleic acids that are analysable from formalin-fixed paraffin-embedded tissue. As a consequence, tissue fixation recommendations are provided in guidance for molecular pathology laboratories [12]. The samples received in this study were too few and widely spread across time and different pathology laboratories for it to be possible to analyse data for variability between pathology labs. However, given that we were able to yield analysable results from several samples over 30 years old, we feel it is reasonable to conclude that length of time since sampling should not be a reason not to undertake deceased index case analysis.

Although the test failure rate was significant, it is important to note that as the analysis took place in a clinical laboratory, in line with validation data and to guard against false positives arising from formalin artefacts, strict threshold criteria were applied for clinical analysis to take place. Analyses that did not reach these thresholds were failed and samples that repeatedly failed were classed as poorly performing samples. The successful analyses were of high quality.

As the aim of this testing is to identify germline variants, it is important that the tissue tested contains non-neoplastic tissue, to avoid detecting any somatic variants present as part of a cancerous tumour. This is an important part of the request process and includes assessment and dissection of FFPE samples by the Consultant Pathologist. This approach has enabled many successful analyses to be achieved, even in tissue samples with a high neoplastic content. Interestingly, in one particular case included in the cohort presented, we accepted shavings containing neoplasia as an exception as there were no non-neoplastic tissue blocks available and the available blocks were not suitable for macrodissection. Testing revealed a class 5 pathogenic variant in the BRCA2 gene which was present in 87% of reads. Given the significant allele fraction of the pathogenic variant, the laboratory felt reasonably confident this variant was likely to have been present in the deceased individual’s germline although the clinical report clearly stated there was a degree of uncertainty as to whether this was a somatic or germline variant and advised caution when interpreting the result. Subsequently, following counselling around this uncertainty, a first degree relative underwent predictive genetic testing and was found to carry the pathogenic variant in their germline. This patient was then able to access high-risk breast MRI screening and risk-reducing surgical options, for which she had not previously been eligible. This also provided the confirmation that the finding in the FFPE sample was reflective of a germline variant in her deceased relative. This particular case highlights that with collaboration between the clinical and laboratory team, this technology can be utilised in cases where only neoplastic tissue samples from a deceased affected relative are available.

Of the total 180 samples tested, the impact on the clinical management of relatives was known or could be inferred in 143 cases. In 54 (38%) of these cases, the results directly impacted the clinical management of living relatives seen by the Clinical Genetics Service. The most significant impact on management was in the 29 cases where a pathogenic/likely pathogenic variant was identified. In these cases, living unaffected family members became eligible to access appropriate screening or to undergo predictive genetic testing when previously they could not. In the 2 years since data collection, in the 17 cases requested by the Manchester Clinical Genetics Service in which a pathogenic/likely pathogenic variant was identified, 44 relatives have undergone predictive genetic testing through our service. In many cases, a negative predictive genetic test result in living relatives unequivocally ruled individuals out of being at an increased cancer risk and reduced the need for additional screening. Conversely, a positive predictive genetic test result in living relatives allowed for more specific management advice to be given. For example, risk-reducing breast and ovarian surgery in women who were found to have a pathogenic/likely pathogenic *BRCA1/2* variant [1].

Of interest, following collection of the data presented in this paper, in an additional case not included in the cohort presented, the NWGLH conducted genetic testing for a known familial pathogenic *BRCA1* variant in an FFPE sample (non-neoplastic tissue) from a deceased individual diagnosed with breast cancer in her 80s. This individual was a distant relative of a family member in whom the pathogenic *BRCA1* variant had first been identified and given her age at diagnosis of breast cancer, (an age common in women in the general population), could have represented a phenocopy. The results of FFPE testing confirmed the presence of the familial *BRCA1* variant in her stored tissue and effectively identified a branch of the family as being at risk and excluded another branch. Therefore, using the results of this single test, it was possible to inform a number of people about their risk and to identify who in the family would benefit from a referral to Clinical Genetics and those for whom a referral was not appropriate. If FFPE testing had not been available, the Clinical Genetics Service could have received referrals for all of these individuals and effectively taken multiple family members who were never at risk through the predictive genetic testing process unnecessarily.

An important point to highlight about the FFPE *BRCA1/2* panel testing is the impact on clinical management of relatives where no pathogenic/likely pathogenic variant was identified. In 24 out of the 83 ‘negative’ panel results, management advice to relatives was adjusted following the result. In many cases, even taking the sensitivity of testing into account, it was possible to offer greater reassurance to some individuals with regard to their breast cancer risk, often impacting screening recommendations. In England, women in the general population are offered three-yearly mammography from the age of 50 through the National Breast Screening Programme [18]. Those at higher risk due to family history (or genetic status) are eligible for additional screening. The frequency and type of screening offered is dependent on whether they meet the moderate risk, high risk or very high risk criteria [1]. For some women, the results of FFPE *BRCA1/2* testing in their deceased relative meant their lifetime risk of breast cancer assessment was reduced to either ‘moderate’ risk or ‘population’ risk and the recommendations for the level of breast screening they were eligible for was adjusted appropriately. In these cases, a negative test result in the affected relative’s FFPE tissue provided more reassurance than if unaffected (or indirect) testing had been carried out in them. We hypothesise that this has a positive impact on these women, but this is something the authors feel would warrant further exploration.

In contrast to the FFPE *BRCA1/2* testing cohort, the impact of a negative panel result on the clinical management of relatives in the FFPE inherited colorectal cancer testing cohort was minimal. In cases where the family history met Amsterdam criteria, indicating a clinical diagnosis of Lynch syndrome in the family [2, 5], or where a clinical diagnosis of FAP had been made, screening recommendations remained as they were before germline FFPE testing was conducted.

A limitation of both the FFPE *BRCA1/2* panel testing and the FFPE inherited colorectal cancer panel testing is that at present, the amplicon-based technology cannot detect large genomic rearrangements (>40 base pairs in size). MLPA copy number analysis is not practicable in these samples and no clinically validated alternative NGS methodology was available. For the *BRCA1/2* genes, it is estimated that large genomic rearrangements account for up to 15% of pathogenic variants, and for the MMR genes it is around 10–25% [19, 20]. Supplementary Table 2 details the NWGLH data on germline large scale genomic rearrangements identified in the *BRCA1/2* genes and the three common MMR genes using blood samples from living affected individuals (i.e., testing done using germline lymphocyte DNA samples). This gives an estimate of the likely percentage of missed variants; an important discussion point when counselling families with negative results. This limitation was highlighted to relatives consenting to the testing, and the sensitivity of the testing was taken into account when providing revised risk figures for families following the results of testing in their affected relative. For those where the family history is particularly suspicious (e.g. Manchester score over 20) MLPA copy number analysis to identify any deletions or duplications is offered to unaffected individuals.

In 6% (9/140) of completed analyses, a Class 3 VUS was identified. From a clinical management perspective, these were viewed in the same way as if a VUS had been identified in lymphocyte DNA from a living affected individual. Prior to testing, relatives were counselled around the possibility of a VUS being identified. Predictive testing was not offered to relatives in these cases, and screening recommendations were unchanged. Such findings are periodically reviewed by the clinical team in case the variant classification changes.

Assessing genetic risk is becoming increasingly important, particularly as genetic testing is becoming mainstreamed in UK healthcare [21]. The benefit of offering germline FFPE genetic testing as part of the UK NHS Clinical Genetics Service is that it fits well into established clinical pathways, with the possibility of this testing now being part of routine care for the patients seen by our Service. This has enabled previous risk assessments to be refined and for management options to be adjusted accordingly.

Prior to this testing being available in the clinical setting, patients with no living affected relatives available for diagnostic testing may not have been offered testing or may have been offered indirect genetic testing. Although in some cases, indirect testing does yield an informative result when a pathogenic/likely pathogenic variant is identified in an unaffected individual, the vast majority of the time it does not. In these cases, the cause of the cancers in the family remains unexplained and relatives are managed on the basis of their family history information. It may be that the cause of cancer in the family is due to a pathogenic/likely pathogenic variant in one of the genes tested although the unaffected individual has not inherited this themselves. In this scenario, the unaffected individual may continue to access high-risk screening and/or risk-reducing surgical options unnecessarily. FFPE testing significantly increases the likelihood of the underlying genetic cause of cancer in a family being identified. A negative predictive genetic test result in an unaffected relative enables a much more accurate assessment of their risk than a negative unaffected genetic test result would have been able to provide (i.e., if FFPE testing had not been possible in their deceased affected relative).

The ability to investigate the underlying genetic cause of cancer in a deceased individual is a powerful tool to offer families as it allows for risk assessments of family histories to be refined which, in turn, impacts management options available. Although analysis failed in 22% of cases, we feel that as the results impacted clinical management of relatives in at least 30% of cases, allowing for better targeted management, the benefits of successful analyses outweigh the disadvantage of the failure rate. An interesting area for future research would be to assess the health economic impact of this testing. We hypothesise that even with the failure rate, it is more cost effective to offer this testing than to offer indirect genetic testing in numerous unaffected relatives. In a publicly funded health system where it is routine practice to manage families rather than individuals in isolation, we feel this testing is incredibly beneficial. In summary, this paper describes the outcome of germline FFPE testing of the *BRCA1/2* genes and a panel of 13 genes linked to inherited colorectal cancer susceptibility in deceased index cases, where previously it had not been possible to offer diagnostic testing to a living affected relative. The clinical data presented indicate that this method is an accurate, informative and valuable tool in the clinical management of patients seen within the Clinical Genetics setting.

## Supplementary information

Supplementary material
