# Supplementary material for: Germline FFPE inherited cancer panel testing in deceased family members: implications for clinical management of unaffected relatives
Source: Eur J Hum Genet. 2021 Mar 2;29(5):861–71. doi: 10.1038/s41431-021-00817-w (PMC8110779; doi:10.1038/s41431-021-00817-w)
Supplement: Supplementary file 1 — Supplementary material [file 41431_2021_817_MOESM1_ESM.pdf]

Supplementary Table 1. Pathogenic and likely pathogenic variants identified.
[Reference sequences used for variant naming are as follows: BRCA1 LRG_292t1 (NM_007294.3), BRCA2 LRG_293t1 (NM_000059.3), MLH1 LRG_216t1 (NM_000249.3), MSH2 LRG_218t1 (NM_000251.2), MSH6 LRG_219t1 (NM_000179.2) & APC LRG_130t1 (NM_000038.4)]

**FFPE panel used**	**Variant identified**	**Variant Class (using ACMG guidelines[16])**
BRCA1/2 panel	BRCA1 c.1961delA p.(Lys654SerfsTer47)	5
	BRCA1 c.2603C>G p.(Ser868Ter)	5
	BRCA1 c.53T>C p.(Met18Thr)	5
	BRCA1 c.5503C>T p.(Arg1835Ter)	5
	BRCA2 c.1041dupA p.(Val348SerfsTer10)	5
	BRCA2 c.4638delT p.(Phe1546LeufsTer22)	5
	BRCA2 c.6065C>G p(Ser2022Ter)	5
	BRCA2 c.8904delC (Val2969CysfsTer7)	5
	BRCA2 c.6275_6276delTT p.(Leu2092ProfsTer7)	5
	BRCA2 c.6938-1G>A	5
	BRCA1 c.3400G>T p.(Glu1134Ter)	5
	BRCA1 c.4721delA p.(Asp1574AlafsTer27)	5
	BRCA1 c.4963T>C p.(Ser1655Pro)	5
	BRCA2 c.3680_3681delTG p.(Leu1227GlnfsTer5)	5
	BRCA1 c.1012A>T p.(Lys338Ter)	5
	BRCA1 c.3400G>T p.(Glu1134Ter)	5
	BRCA1 c.4065_4068delTCAA p.(Asn1355LysfsTer10)	5
	BRCA1 c.5406+5G>C	4
	BRCA2 c.517-2A>G	5
	BRCA2 c.67+2T>C	5
Inherited colorectal panel	MSH2 c.425_429dupCAGCT	5
	APC c.1863_1866delTTAC p.(Tyr622GlyfsTer7)	5
	MSH6 c.3724_3726delCGT p.(Arg1242del)	4
	MSH2 c. 1700_1704delAAACA p.(Lys567ArgfsTer3)	5
	MLH1 c.1570_1592delinsTTCTCC	5
	MLH1 c.1943C>T p.(Pro648Leu)	5
	MSH6 c.3247delG	5
	MSH2 c.2634+1G>T	5
	MLH1 c.350C>T p.(Thr117Met)	5

Supplementary Table 2. Current detection rate for large genomic rearrangements in the BRCA1/2 and MMR genes tested using blood samples in living affected patients who had diagnostic testing through the NW GLH

Gene	Total number of variants identified	Number of variants identified by MLPA	Percentage of variants identified by MLPA
BRCA1	664	142	21.39%
BRCA2	666	36	5.41%
MLH1	147	18	12.24%
MSH2	184	46	25.00%
MSH6	66	1	1.52%
